# No association between genetic ancestry and exome sequencing-based diagnosis of inborn errors of metabolism

**DOI:** 10.1038/s41525-026-00562-3

**Published:** 2026-03-27

**Authors:** J. Najera, Y. Mavura, A. Adhikari, M. Kvale, R. C. Gallagher, N. Risch

**Affiliations:** 1 Institute for Human Genetics, University of California, San Francisco, San Francisco, CA USA; 2 Illumina Corporation, San Diego, CA USA; 3 Department of Pediatrics, University of California, San Francisco, San Francisco, CA USA

**Keywords:** Metabolic disorders, Paediatric research, Genetic testing

## Abstract

Inborn errors of metabolism (IEMs) are severe genetic disorders caused by disruptions in metabolic pathways, frequently presenting in early life. Exome (ES) and genome (GS) sequencing have revolutionized the diagnostic approach for Mendelian disorders. However, most studies evaluating the diagnostic yield of ES have focused on predominantly European ancestry populations, leaving significant gaps in understanding its efficacy across diverse ancestries. We evaluated the diagnostic yield of ES in a cohort of 845 newborns with clinically diagnosed IEMs through the California Department of Public Health’s Genetic Disease Screening Program (GDSP) and its relationship to genetic ancestry. Over 55% of this ancestrally diverse cohort was maternally reported as non-white/European. By estimating genetic ancestry from exome sequencing data, we assessed its relationship with ES diagnostic outcomes. Diagnostic yield by ES did not significantly differ by any genetic ancestry, supporting its equitable performance across all ancestries among these largely recessive disorders. Higher consanguinity coefficients were associated with increased homozygosity among exome-positive cases, without affecting diagnostic yield across genetic ancestries.

## Introduction

Inborn errors of metabolism (IEMs) are a diverse group of genetic disorders caused by defects in metabolic pathways, often manifesting early in life with symptoms such as failure to thrive, developmental delays, and multi-system complications due to the accumulation of toxic metabolites or energy deficiencies. Each year in the United States, newborn screening identifies approximately 1 in 320 newborns—around 12,500 of the nearly 4 million annual births—with a potentially severe or life-threatening condition before clinical symptoms appear, with 10% of these cases involving IEMs^1^.

The advent of exome sequencing (ES) and genome sequencing (GS) has revolutionized the diagnosis of rare Mendelian disorders, providing unparalleled speed and accuracy in uncovering their molecular causes and reshaping the field of genetic testing in both clinical and research settings. ES and GS are increasingly being considered as first-line diagnostic tools for certain genetic conditions where early detection and treatment are essential for improving outcomes^2–4^. However, most studies evaluating the diagnostic yield of ES and GS have focused predominantly on populations of European ancestry, leaving significant gaps in our understanding of its performance across diverse genetic backgrounds^5,6^.

The likelihood of obtaining a positive genetic diagnosis, known as diagnostic yield, may be influenced by various factors, such as the patient’s clinical phenotype, degree of consanguinity, family history, and the number of family members sequenced alongside the proband^5,7,8^. However, the role of genetic ancestry in influencing diagnostic yield remains unclear, as it is intertwined with social challenges such as the underrepresentation of non-European populations in clinical genomic reference databases or possibly the availability of parents for testing in addition to the proband. It has been suggested that these gaps lead to lower diagnostic yields^5,9–11^.

To investigate the influence of genetic ancestry on diagnostic yield of IEMs, we analyzed the diagnostic outcomes of ES in a cohort of newborns clinically diagnosed with IEMs through the California Department of Public Health’s newborn screening program. Our previous study of this cohort compared ES as a primary screening tool to analyte screening by tandem mass spectrometry (MS/MS) and found that the sensitivity and specificity of ES were inferior to MS/MS. However, ES demonstrated utility in reducing false-positive callouts and served as a valuable secondary follow-up test after a positive MS/MS screen^12^. By further examining this ancestrally diverse cohort, we aimed to assess whether diagnostic yield from ES varied across genetic ancestries and to explore the potential role of consanguinity in shaping diagnostic outcomes.

Notably, over 55% of our cases had a maternal report of race/ethnicity classified as non-White, including African American, East Asian, South Asian, Middle Eastern and Latino(a), providing a unique opportunity to evaluate ES diagnostic yield across a broad range of ethnicities/ancestries. While previous studies have examined diagnostic yield in ancestrally diverse populations, they have typically included a mix of inheritance patterns, encompassing both autosomal dominant and recessive disorders, with the majority of diagnosed cases typically due to autosomal dominant mechanisms^5,9^. In contrast, our study takes a unique approach by focusing almost exclusively on recessive disorders. To our knowledge, no study has specifically examined the impact of genetic ancestry on diagnostic yields for IEMs using exome sequencing, though prior research has explored this relationship using panel-based genetic testing in a very small cohort of IEM patients^11^. Our results may also have implications for a broader array of recessive disorders.

## Results

### Race, ethnicity, and genetic ancestry of NBseq newborns

The results of PCA are detailed in Supplementary Figs. S1 and S2. In Fig. S1, the distribution of newborns extends primarily along the first principal component (PC1), capturing the largest component of genetic variance. The primary differentiation along PC1 is the delineation of African ancestry from other groups. The second principal component (PC2) discriminates European (blue) and Middle Eastern (yellow) ancestry from East Asian (tan) and Native American (purple) ancestry, arraying them vertically from top to bottom. The NBseq samples aggregate heavily along PC2, suggesting predominance of European, East Asian, and/or Native American genetic ancestry. East Asian and Native American genetic ancestry are not distinguished in these two PCs. We also observe a moderate number of NBseq samples on the line between the African and European ancestry groups. These are individuals identified as African American who have mixed African/European genetic ancestry.

In Fig. S2, East Asian ancestry is clearly separated from Native American ancestry, and the Pacific Islander/Oceanian (orange) ancestry, in both PC3 and PC4. In this figure, we can see a dominance of NBseq samples along a line between European and Native American groups, suggesting many individuals identified as Hispanic/Latino. We also see a cluster of NBseq samples on top of the HGDP East Asians but no cluster with the Pacific Islanders/Oceanians.

From the genetic admixture analysis, the average genetic ancestry for the entire 845 NBSeq cases was as follows: 7.1% African, 11.0% East Asian, 44.8% European, 8.8% Middle Eastern, 21.5% Native American, and 6.4% South Asian.

**Fig. 1:**
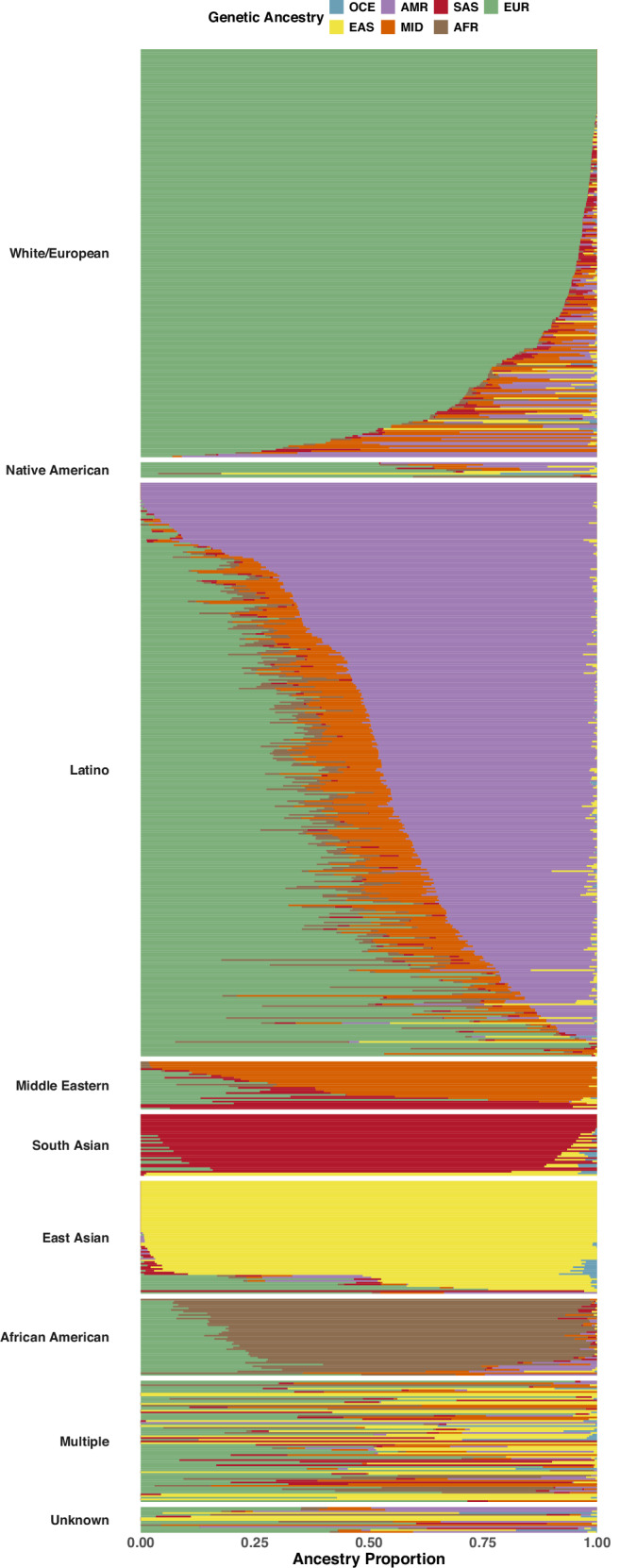
Correspondence between estimated genetic ancestry proportions and self-reported race/ethnicity. Horizontal lines represent individual-level genetic ancestry estimates, with colors indicating different ancestral components. Group labels on the left correspond to self-identified racial/ethnic categories.

The admixture proportions in Fig. 1 provide a comparative analysis of the race/ethnicity of the infant reported by the mother against the genetic ancestry estimated from the ES data.

Individuals identified as African American predominantly exhibited a mixture of African and European genetic ancestries. Participants reported as South Asian displayed primarily Central/South Asian genetic ancestry. For those identified as East Asian, the genetic ancestry was almost exclusively East Asian. However, an exception was noted among those who were identified as Filipino, some of whom showed a mixture of European and East Asian ancestries.

Participants who were reported as Middle Eastern ethnicity demonstrated a blend of European and Middle Eastern genetic ancestries. Those identified as non-Hispanic White were predominantly of European genetic ancestry with minor Middle Eastern genetic contributions. Participants identifying as Hispanic exhibited the most variation in genetic ancestry, with some showing predominantly European genetic ancestry, while others displayed a significant presence of Native American ancestry, alongside consistent but minor traces of Middle Eastern and African ancestries.

Individuals identified as Native American had, on average, a greater proportion of European and less Native American genetic ancestry compared to those identified as Hispanic. Lastly, those reported as mixed race showed European genetic ancestry, complemented by other genetic backgrounds.

### Genetic ancestry and diagnostic yield

We defined diagnostic yield as the proportion of total IEM cases that received an exome diagnosis (i.e., homozygous for or with two different reportable variants in an autosomal gene or a single reportable variant in males in an X-linked gene consistent with their identified diagnosis). Those with one or no called variants in a corresponding autosomal gene or 0 variants in an X-linked gene for their disorder were considered exome negative.

**Fig. 2:**
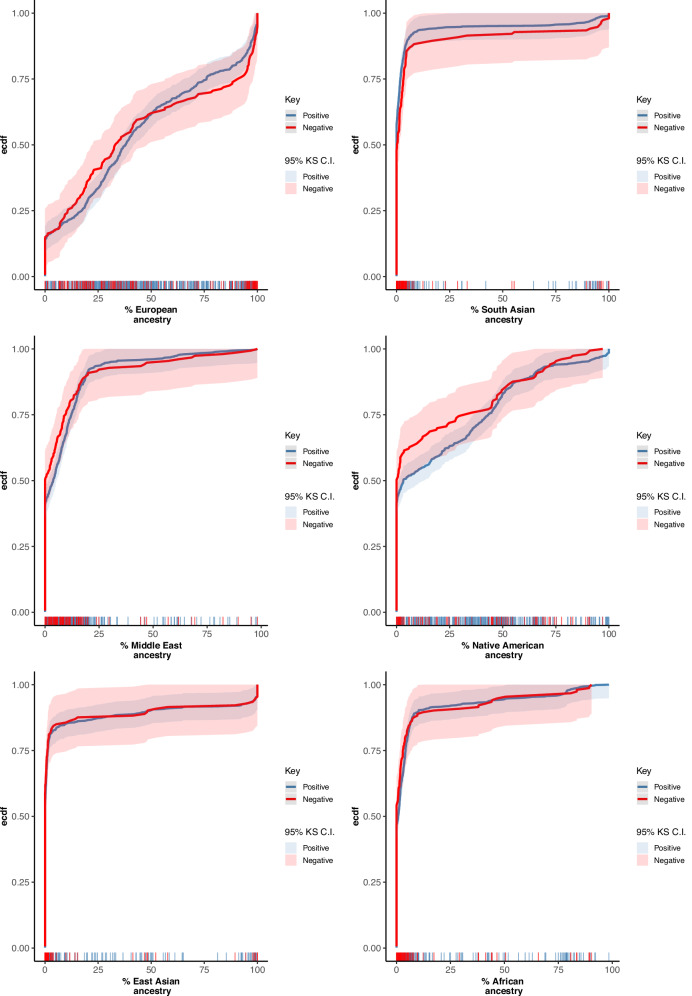
Distribution of genetic ancestry proportions in exome-positive and exome-negative cases. Empirical cumulative distribution function (eCDF) curves comparing the distribution of estimated genetic ancestry proportions between exome-positive and exome-negative newborns for each ancestry group. Bars along the *x*-axis indicate individual ancestry proportions for exome-positive and exome-negative cases. Differences between groups were evaluated using the Kolmogorov–Smirnov test.

The distributions of estimated genetic ancestries of the newborns (Fig. 2) displayed varying degrees of discontinuity, depending on the genetic ancestry group. The Native American and European ancestries were more continuous while the African, Middle Eastern, East Asian, and South Asian were more discrete. The Kolmogorov–Smirnov (KS) test (Table 1) revealed a nominally significant discrepancy in the distributions of exome-positive versus exome-negative cases of South Asian and Native American ancestries (reduced diagnostic yield associated with South Asian genetic ancestry, increased diagnostic yield associated with Native American genetic ancestry), however neither of these was significant after adjustment for multiple testing.

**Table 1:** Kolmogorov–Smirnov test results

Ancestry	D statistic	*P*-value
EUR	0.09	0.30
SAS	0.14	0.01
MID	0.10	0.13
EAS	0.06	0.75
AMR	0.12	0.05
AFR	0.10	0.20

**Fig. 3:**
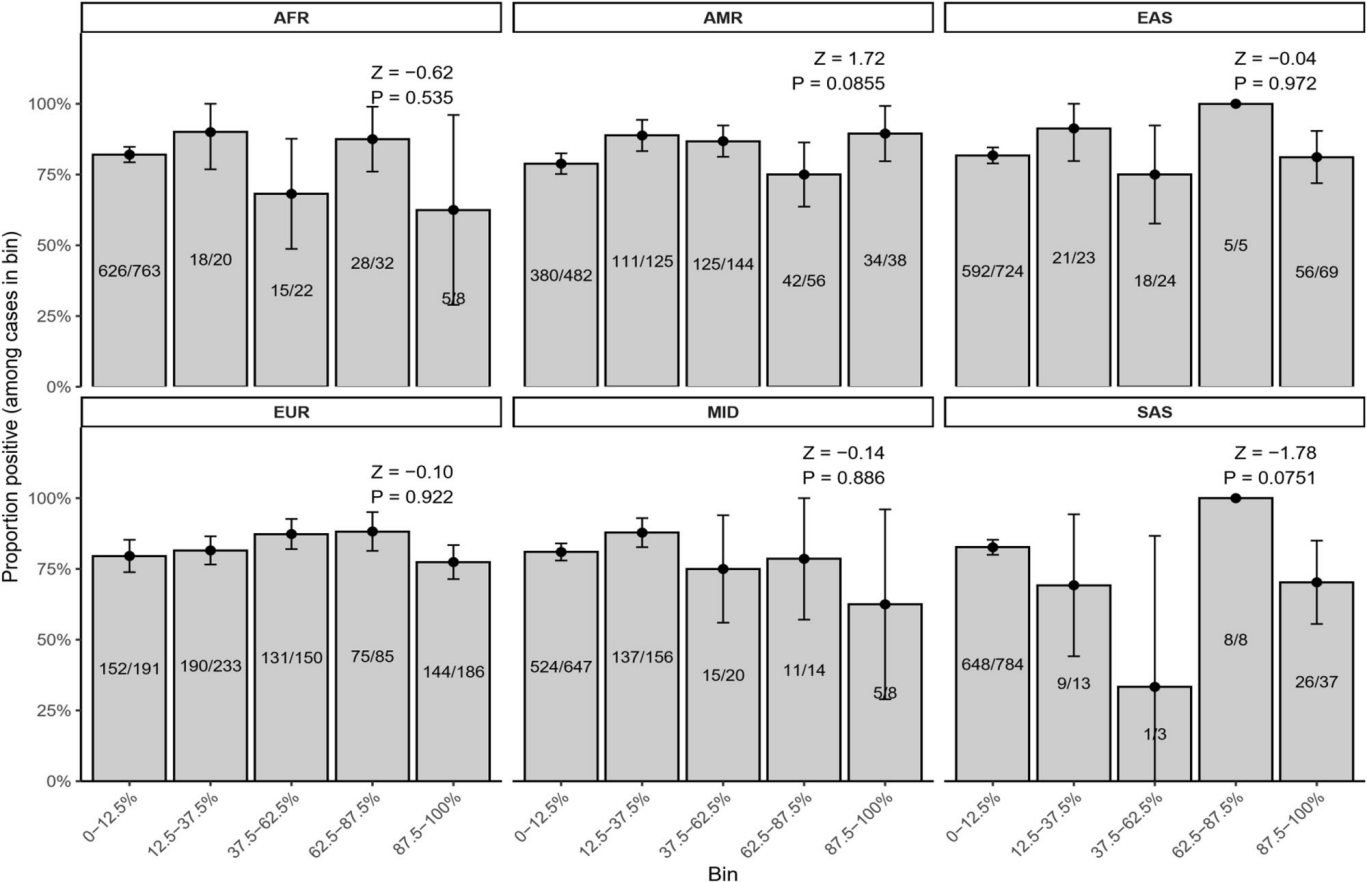
Cochran–Armitage trend test of exome diagnostic yield across genetic ancestry bins. For each ancestry, newborns were grouped into bins of estimated genetic ancestry proportion, and Cochran–Armitage trend tests were used to assess linear trends in exome diagnostic yield across bins. Points represent test statistics for each ancestry, and error bars or accompanying labels, if shown, indicate the corresponding significance values. Positive values indicate increasing diagnostic yield with greater ancestry proportion, whereas negative values indicate decreasing diagnostic yield.

**Fig. 4:**
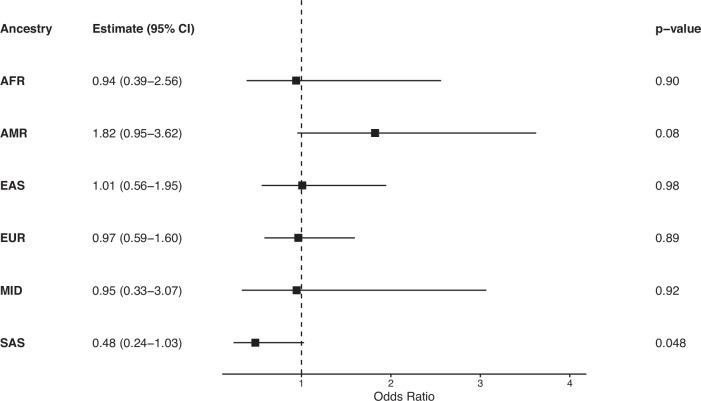
Logistic regression analysis of exome diagnostic outcome by genetic ancestry. Results from logistic regression models evaluating the association between estimated genetic ancestry proportion and exome-positive diagnostic outcome. Points represent odds ratio estimates for each ancestry, and horizontal lines indicate 95% confidence intervals. The vertical reference line at an odds ratio of 1 indicates no association between ancestry proportion and diagnostic outcome.

The *z*-scores and *p*-values for the Cochran–Armitage trend tests (see “Methods”) are detailed in Fig. 3. Consistent with the KS test results, a suggestive negative correlation between South Asian ancestry and diagnostic yield and a trend towards increased diagnostic yield with Native American ancestry were observed; however, neither was significant after correcting for multiple testing. These observations were also consistent with the results of logistic regression analyzes, which also showed a suggestive negative association of South Asian and positive association of Native American genetic ancestry with exome diagnostic outcome (Fig. 4). However, as before, no associations were significant after multiple testing corrections.

### Consanguinity coefficient and diagnostic yield

The *F* coefficient among the 692 cases that demonstrated positive ES diagnostic outcomes suggested varying degrees of consanguinity. Specifically, 154 (18.5%) of these cases exhibited an *F* coefficient greater than 0.015625, which corresponds to parental genetic relatedness typical of second cousins, 70 (8.4%) cases displayed an *F* coefficient exceeding 0.03125, aligning with the consanguinity level observed between parents that are first cousins once removed, and 29 (3.5%) cases had an *F* coefficient greater than 0.0625, indicative of parental consanguinity equivalent to that of first cousins. *F*-scores differed significantly across ancestry groups (Kruskal–Wallis *p* < 2.2 × 10⁻¹⁶), with the South Asian group showing higher median *F* values relative to other ancestries (Table S1).

**Fig. 5:**
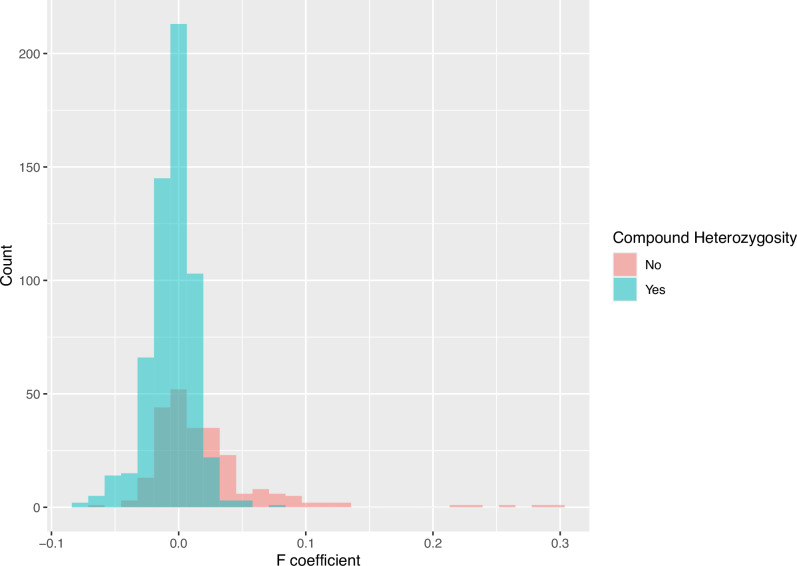
Distribution of consanguinity coefficients among exome-positive cases by zygosity class. Histograms showing the distribution of the F coefficient among exome-positive newborns stratified by homozygous and compound heterozygous diagnostic outcomes. The *x*-axis shows the estimated F coefficient and the *y*-axis shows the number of individuals.

We examined whether higher *F* coefficients were associated with newborns that were homozygous vs compound heterozygous for exome positive outcomes using logistic regression, which was highly significant with a *p*-value less than 0.0001 (Table 2), strongly suggesting that those with homozygous pathogenic alleles were often due to consanguinity. On average, a 0.01 increase in *F* coefficient was associated with 50% higher odds of homozygosity compared to compound heterozygosity across all ancestries (OR = 1.50, 95% CI: 1.38–1.63). We also created histograms of the *F* coefficient for homozygous versus compound heterozygous positive cases (Fig. 5). Here we observe that the distribution of *F* values for the homozygous cases is multimodal and skewed, with modes around 0.03125 and 0.0625, the average *F* values for children of mating of first cousins once removed and first cousins, respectively, and a few more extreme values, corresponding to closer and possibly more complex consanguinity patterns. By contrast, the distribution for the compound heterozygotes is roughly normally distributed with a mode at 0 and no additional modes.

**Table 2:** Results from a logistic regression model assessing the association between the *F* coefficient and homozygosity versus compound heterozygosity by race/ethnicity

Race/ Ethnicities	Estimate	Standard error	*P*-value	Odds ratio (per 0.01 increase in *F* coefficient)	Confidence lower bound	Confidence upper bound
All Ancestries	40.28	4.36	2.40 × 10⁻²⁰	1.50	1.38	1.63
Hispanic	45.12	7.25	4.86 × 10⁻¹⁰	1.57	1.37	1.82
White	16.83	10.30	1.02 × 10⁻¹	1.18	0.97	1.48
Black	15.44	21.83	4.79 × 10⁻¹	1.17	0.77	1.85
East Asian	169.81	51.74	1.03 × 10⁻³	5.46	2.43	20.13
South Asian	94.49	34.07	5.55 × 10⁻³	2.57	1.51	5.93

Additionally, we explored potential variation in the association of *F* coefficients with homozygosity across different racial/ethnic groups by examining histograms stratified by race/ethnicity: Hispanic/Latino, White/European, African American or Black, South Asian, and East Asian (Table 2, and Fig. S4).

Patterns in *F* values differ by race/ethnicity, with the strongest associations observed in South Asians and East Asians. A 0.01 increase in *F* coefficient was associated with a 157% increase in the odds of homozygosity for South Asians (OR = 2.57, 95% CI: 1.51–5.93) and a 446% increase for East Asians (OR = 5.46, 95% CI: 2.43–20.13), though small sample sizes in these groups may introduce variability in the estimates and large confidence intervals.

In Hispanic/Latino individuals, a 0.01 increase in *F* coefficient was associated with a 57% increase in the odds of homozygosity (OR = 1.57, 95% CI: 1.37–1.82), consistent with the bimodal distribution of *F* values for homozygotes observed in this population (Fig. S4).

In White and Black individuals, the association between *F* coefficient and homozygosity was weaker, with OR = 1.18 (95% CI: 0.97–1.48) for Whites and OR = 1.17 (95% CI: 0.77–1.85) for Blacks. The confidence intervals for these groups include 1.0, suggesting that the relationship between consanguinity and homozygosity is weaker in these populations.

### Ancestry variation with curated variants only

As described in “Methods”, we stratified our ES-diagnosed cases into those whose diagnosis was based solely on previously curated variants (*N* = 444) versus those whose diagnosis included non-curated variants (*N* = 251). We then compared average ancestries for each of 6 ancestries between these two subgroups by *t*-tests (Table S2). Here we observed a mild but statistically significant increase in Eur genetic ancestry (*P* < 0.01) and decrease in Amr genetic ancestry (*P* < 0.05) in the curated versus non-curated subgroups, while there was no difference for any of the other 4 genetic ancestries. This may be due to previously non-curated founder variants among Native Americans, manifesting in the Latinx cases. However, as described above, including all rare protein-altering variants led to no difference in diagnostic yield for these ancestries.

## Discussion

In the analysis of diagnostic yield by exome sequencing of a cohort of ancestrally diverse newborns identified to have an IEM after positive newborn screening by MS/MS, we observed no reduction in the overall diagnostic yield associated with any of the non-European genetic ancestries (African, Native American, East Asian, European, Middle Eastern, South Asian).

Our findings, which suggest that the diagnostic yield from exome sequencing does not vary significantly among European and non-European genetic ancestries, align with some prior studies that examined clinical cases across various modes of inheritance in pediatric and prenatal cohorts^9^. In those studies, most cases were autosomal dominant and included many de novo variants, although recessive cases were also present. Low allele frequencies for pathogenic/likely pathogenic (P/LP) variants were a consistent observation. By contrast, our study, which focused almost exclusively on autosomal recessive inheritance for IEMs, included some variants that were not extremely rare (allele frequency >0.001), likely due to founder effects. Despite these differences in variant frequency, we still detected no disparity in diagnostic yield by genetic ancestry, even in this recessive disease setting with more common variants.

In contrast, other research has highlighted disparities in genetic testing diagnostic yields across racial groups. For example, studies using predefined genetic panels for sensorineural hearing loss reported that Hispanic and Black children were less likely to receive a definitive genetic diagnosis compared to White and Asian children^10^. However, these disparities were resolved when an agnostic in silico method was employed to evaluate the effect of variants on predicted protein function, revealing that the disparities were driven by inequities in P/LP variants that had been included in pre-defined panels, likely due to under-inclusion of variants rare in Europeans but more common in other ancestries^10^. In our study, variant annotation was broad and genomic, encompassing all protein-altering, rare, and novel variants, and did not depend on prior classification as pathogenic (P) or likely pathogenic (LP), with conclusions similar to those in the hearing loss study. As a consequence, referral to reference databases of P/LP variants, which might be biased towards European ancestry, was not an issue in our setting, because our broadest category of variant annotation, which included all protein-altering variants with MAF < 0.5%, did not depend on any such reference databases of previously identified P/LP variants. In contrast, the Deciphering Developmental Disorders project found significantly lower diagnostic yields for probands of non-European ancestry compared to European ancestry^5^. While individuals of non-European ancestry exhibited a greater number of reported variants compared to those of European ancestry, a smaller fraction of these variants were classified as “pathogenic” or “likely pathogenic,” particularly among singleton cases in which parents were not also sequenced. This study, which used an extensive variant-detection pipeline rather than a predefined genetic panel, demonstrated that disparities in diagnostic yield persisted even when applying an agnostic approach for variant classification. These disparities were partly attributed to the reduced likelihood of being recruited as part of a family trio, which was differential by race/ethnicity. Trio-based analysis helps clarify variant pathogenicity by identifying inheritance patterns, phase, and de novo mutations, reducing the likelihood of variants being classified as uncertain.

A key distinction of the NBseq project from some prior large-scale studies is its predominant focus on recessive disorders. Recessive disorders often involve common founder pathogenic variants, which can potentially introduce differences when comparing diagnostic yields across ancestry groups. Founder mutations may disproportionately occur in certain populations for specific autosomal recessive diseases, potentially resulting in an overrepresentation of diagnostic results in those populations. Unlike studies that examined a mix of autosomal dominant and recessive diseases, focusing exclusively on recessive disorders could increase the chance of potential differences associated with founder mutations. Nonetheless, we observed no differences in diagnostic yield of the exome sequencing among genetic ancestries, providing reassurance for its equitable application in this setting of ES-based IEM diagnosis.

NBseq exclusively sequenced newborns and did not include parental sequencing data. While the absence of parental information limits the ability to phase variants for compound heterozygosity, it also eliminates potential confounding introduced by differences in family trio availability. In our study, in addition to homozygosity due to founder mutations, degrees of consanguinity in certain populations increased the likelihood of homozygous variants, which do not require phasing, among positive cases. However, this did not lead to overall differences in diagnostic yield.

As expected, we identified a significant link between higher consanguinity coefficients and the presence of homozygous pathogenic variants. Notably, 18.5% of positive diagnostic outcomes corresponded to levels of consanguinity akin to second cousins or closer. Logistic regression confirmed a significant positive correlation between consanguinity and homozygosity, with homozygous variants being more prevalent in cases of higher consanguinity (*p* < 0.0001).

Further analyses exploring racial/ethnic differences revealed distinct patterns in the distribution of *F* coefficients. Initial stratified histograms suggested higher rates of consanguinity among South Asian and East Asian cases. In Hispanic/Latino cases, we also observed a subset of cases with increased *F* values for homozygotes, while African Americans and Whites showed no significant differences except for occasional outliers. To formally evaluate these differences, we conducted a Kruskal–Wallis test, which confirmed that *F*-scores varied significantly across ancestry groups. Median *F* values supported this pattern: South Asian cases showed the highest median F (0.0157), consistent with elevated background levels of consanguinity, whereas European (–0.0026) and African (–0.0195) cases exhibited the lowest *F* values (Table S1). These differences may also reflect the frequency of variants involved, as consanguinity is expected to be greatest when the disease variants are least frequent.

While this study focused on a specific subset of largely autosomal recessive disorders, specifically inborn errors of intermediary metabolism diagnosed after a positive analyte newborn screening test, our results may have broader implications for other recessive disorders. As opposed to dominant disorders, which can have a variety of functional mechanisms, such as gain or altered function or haploinsufficiency, recessive disorders are generally due to loss of function homozygous or compound heterozygous variants. As such, annotating such variants may be more straightforward, although complexity still remains. However, in terms of generalization, it is important to distinguish our setting from others. In our situation, we focused on characterizing genetic variants as P/LP within a single or a small number of genes associated with specific, well-characterized IEM disorders—and not the entire exome. This enabled a broad annotation algorithm of variants that included all rare protein-altering changes that did not depend on their presence in databases. When we compared cases that only included previously curated variants to those that included non-curated variants, we found a modest difference in European and Native American genetic ancestries (but not others – notably including African genetic ancestry). Thus, in a setting where the entire exome is a candidate for a previously undiagnosed or unrecognized recessive disorder, such a broad inclusion of variants is not practical, and greater variant annotation/curation is required; hence, some ancestry differences may remain, and this question clearly merits further investigation. As more disease-causing genetic variants from non-European ancestries are identified, we may expect this situation also to evolve over time.

Although this study represents one of the largest ancestrally diverse evaluations of exome sequencing diagnostic yield for IEMs to date, the absolute sample size is constrained by the rarity of these disorders. Replication in larger cohorts and across additional recessive disease groups will be important to confirm the robustness and generalizability of these findings. Future studies leveraging independent newborn screening programs or multi-site collaborations will be particularly informative.

## Methods

### Participant demographics and exome sequencing

This study was conducted as part of the Newborn Sequencing (NBSeq) project, which evaluated the use of DNA sequencing in newborn screening using de-identified residual dried blood spots (DBSs) collected by the California Department of Public Health (CDPH) Genetic Disease Screening Program. All procedures involving human participants were conducted in accordance with the ethical standards of the relevant institutional and/or national research committees and with the Declaration of Helsinki. The study was reviewed and approved by the Committee for the Protection of Human Subjects of the State of California (Project No. 14-07-1650). Use of DBSs for research was granted under a waiver of informed consent, in accordance with CDPH policies and the Biospecimen/Data Use and Confidentiality Agreement. Details of this study population, referred to as the Newborn Sequencing (NBSeq) study, and the ES performed have been previously published.¹² In this study, ES was utilized to analyze 1416 DBSs derived from an extensive neonatal screening program conducted by the GDSP of the California Department of Public Health (CDPH), which initially screened nearly 4.5 million neonates. The DBSs were used to create 1190 high-quality exomes, representing 845 individuals diagnosed with IEMs and 345 individuals who, despite initial positive identification through multiplex MS/MS, were subsequently classified as unaffected (i.e., MS/MS false positives) based on subsequent testing.

The demographics of true positive cases showed a reported gender distribution of 42.1% females (477), 56.4% males (356), and 1.4% unknown (12). The genetic analysis was targeted at an ‘exome slice’ of the ES data, comprising 78 genes, which are associated with the 48 IEMs screened by the GDSP. Out of the 845 true positive cases, 692 exhibited positive findings on exome sequencing, whereas 153 true positive cases did not demonstrate exome-based evidence of the expected IEMs. Further details of the ES and approach to variant annotation and diagnostic outcomes are provided by Adhikari et al.^12^.

### Maternal reports of race/ethnicity of newborns

The mothers of affected newborns responded to questions about their demographic background on a health survey at the time of specimen collection and specifically the race/ethnicity of their newborn^13^. Regarding race, ethnicity, and nationality, the mother could choose among 18 distinct race/ethnicity categories that were present on the NBS requisition form. Two of these were collapsed into others, giving 16 different, distinct categories:

A. *Southeast Asian:* Any combination of Southeast Asian categories (Cambodian, Laotian, Vietnamese, other Southeast Asian)
B. *Black:* Black only
C. *Multiple Asian:* Any combination of two or more Asian categories (Asian East Indian, Cambodian, Chinese, Filipino, Japanese, Korean, Laotian, Middle Eastern, other Southeast Asian, Vietnamese), except those already included in “Southeast Asian”
D. *Native American:* Native American only or Native American and any non-Asian category
E. *Middle Eastern:* Middle Eastern only
F. *Asian East-Indian:* Asian East-Indian only
G. *Vietnamese:* Vietnamese only
H. *White:* White only
I. *Multiple:* Any combination of two or more categories not already included in any other group
J. *Filipino:* Filipino only or Filipino and white or Filipino and Hispanic or Filipino, white, and Hispanic
K. *Chinese:* Chinese only
L. *Hispanic:* Hispanic only or Hispanic and white
M. *Laotian:* Laotian only
N. *Japanese:* Japanese only
O. *Pacific Islander:* Any combination of Pacific Islander categories (Hawaiian, Guamanian, Samoan)
P. *Korean:* Korean only
Q. *Unknown:* No race, ethnicity, or nationality provided

For the purpose of our analysis, we collapsed these into the following 9 categories:

- Latino (LT) — based on category (L)
- White/European (EU) — based on category (H)
- African American or Black (AF) — based on category (B)
- South Asian (SAS) — based on category (F)
- East Asian (EAS) — based on categories (A, C, G, J, K, M, N, P)
- Middle Eastern (ME) — based on category (E)
- Native American (NA) – based on category (D)
- Pacific Islander (OCE) — based on category (O)
- Multiple (MLT) — based on category (I)
- Unknown (U) — based on category (Q)

### Genetic ancestry and consanguinity analysis

Variants in all VCF files with sequencing depth at or below 10 (DP ≤ 10) or genotype quality equal to or less than 20 (GQ ≤ 20) were filtered out using GATK (Genome Analysis Toolkit)^14^. The VCF files were then lifted over from human genome reference version GRCh37 to GRCh38 using the Picard tool in GATK suite of tools^14^. Human Genome Diversity Panel (HGDP) whole genome sequencing samples from the GnomAD v3 joint call set were used as the reference for genetic ancestry and admixture estimation (*N* = 828 unrelated individuals), following the gnomAD v3 ancestry inference framework^15–17^. The HGDP samples were all mapped to the GRCh38 reference sequence. Ancestry analysis was done on a total of 1289 individuals that had genetic data in NBseq even though they were not all part of the case yield analysis.^12^

High-performance markers were selected from the HGDP and NBseq data for downstream genetic ancestry, admixture, relatedness, and consanguinity analysis using the following criteria:

1) Restriction of markers in the HGDP dataset to exome sequenced regions in the NBseq dataset. This was conducted using bcftools^18^.

2) MAF ≥ 0.05 in any of 7 supergroups in gnomAD HGDP unrelated individuals:

(i) African, (ii) Native American, (iii) South Asian, (iv) East Asian, (v) European, (vi) Middle Eastern, (vii) Oceanian.

3) Only biallelic, autosomal SNPs, with a call rate >95% in exome regions that were sequenced (This was done in both HGDP and NBseq cohorts separately). The resulting markers in the HGDP cohort (*N* = 1,853,287) were intersected with markers from the NBseq cohort sample VCFs, resulting in *N* = 127,638 markers. Variants in regions known to affect principal components (PCs) (HLA region on chromosome 6p, inversion on chromosome 8p23 and inversion on chr 17q21, GRCh38 build) were removed resulting in 120,872 markers after filtering. After linkage disequilibrium pruning (*r*^2^ threshold of 0.5 in a 5 MB window), 88,661 high-performance markers for principal components analysis and genetic admixture analysis remained.

Individual genetic ancestry admixture proportions were estimated using the ADMIXTURE software package using the set of 88,661 exome-wide markers^19^. A supervised approach, whereby unrelated individuals in (K) reference populations are assumed to have 100% reference genetic ancestry, was utilized to estimate the individual genetic admixture proportions in individuals of the NBseq cohort. We created *K* = 7 reference continental/subcontinental ancestral populations from the HGDP individuals, based on the literature^20^. The 7 reference populations were: African–Afr (Yoruba, Mandenka *N* = 40), Native American–Amr (Colombia, Karitiana, Surui, Pima, *N* = 40); East Asian–Eas (Han, Japanese *N* = 40); Middle Eastern–Mid (Druze, Palestinian, Bedouin, *N* = 40); European-Eur (French, Orcadian, Tuscan, Sardinian, *N* = 40); Oceanian–Oce (Papuan, Melanesian *N* = 30); South Asian–Sas (Pathan, Sindhi, *N* = 39). The genetic ancestry admixture proportions were visualized using ggplot2^21^.

Principal components analysis (PCA) was performed on the HGDP samples using the SmartPca program, part of the EIGENSOFT4.2 software package using the same 88,661 markers^22^. The NBseq samples were then projected onto the HGDP-derived PCs to facilitate geographic/ancestral interpretation of the NBSeq participants’ PCs.

Genetic kinship between NBseq participants was estimated using PC-Relate^23^. Genetic ancestry was controlled by using the first 8 PCs from PCA in PC-Relate to estimate only recent genetic relatedness due to family structure. Linkage disequilibrium (LD) pruning (0.1 kb in a 1000 kb window) was done to select a set of independent SNPs for the relatedness analysis including the PCA used for control of ancestry. Similarly, consanguinity coefficients for probands were estimated using PC-relate, also controlling for ancestry using the first eight PCs. Consanguinity coefficient (*F*) is the probability that two alleles at a locus in an individual are identical by descent from a common ancestor. Children of 1st cousins have a consanguinity coefficient of 1/16 (0.0625), while of 2nd cousins it is 1/64 (0.0156).

### Annotated variants and classification of cases

Details of variant annotation are described by Adhikari et al.^12^. Briefly, the analysis was restricted to reportable variants within exonic and splice regions (including near-exon intronic regions) of 78 genes associated with 48 IEMs, considering only those variants with a genotype quality (GQ) score >15. All rare protein-altering variants identified through the exome analysis pipeline were annotated as pathogenic to maximize sensitivity for newborn screening (NBS). This classification was based on two arms used to define reportable variants:

The predicted impact arm included all variants with a minor allele frequency (MAF) < 0.5% in both ExAC and the 1000 Genomes Project that met at least one of the following criteria:

- Annotated as protein-altering by varant type (e.g., StopGain, StopLoss, FrameShiftInsert, FrameShiftDelete, SpliceDonor, SpliceAcceptor, NonSynonymous Missense, InFrameDelete, InFrameInsert, StartGain, StartLoss)
- CADD score >23
- Splicing-effect score >0.5 based on the dbscSNV computational meta-prediction tool

The curation arm included variants with autosomal MAF < 0.1% in both databases, annotated as “DM” or “DM?” in HGMD, or as “pathogenic” or “likely pathogenic” in ClinVar with at least one review star. The NBSeq team also manually curated 60 variants with MAF ≥ 0.1% from these sources, identifying 19 as reportable and excluding 41.^12^

In terms of diagnoses based on the variant annotation results, exome positive cases were identified as individuals homozygous for a reported variant, or presumed compound heterozygous for two reported variants in an autosomal recessive gene matching their identified diagnosis as assessed by a medical geneticist based on additional follow-up testing and clinical evaluation or as a single reported variant for the X-linked disorders, and exome negative cases were identified as individuals heterozygous or with 0 reported variants in a matching autosomal recessive gene or with 0 reported variants for an X-linked gene.

### Statistical analyses

The analysis aimed to compare the distribution of genetic ancestries among exome-positive and exome-negative cases. A Kolmogorov–Smirnov test was employed to evaluate differences in empirical cumulative distribution functions of estimated genetic ancestries among the exome-positive and exome-negative cases. Due to the non-normal and discontinuous nature of the genetic ancestry distributions, five bins were created: 0–12.5%; >12.5–37.5%; >37.5–62.5%; >62.5–87.5%; >87.5–100%. These bins reflected ranges designed to correspond to genetic ancestry proportions that reflect having zero to four grandparents from different ancestral genetic backgrounds. Non-parametric Cochran–Armitage trend tests were conducted to assess linear trends between ES diagnostic yield and estimated genetic ancestry within these bins. In addition to the Kolmogorov–Smirnov and Cochran–Armitage trend tests, logistic regression was performed with ES diagnosis as the outcome and genetic ancestry as the predictor variable.

For the consanguinity analysis comparing coefficients of consanguinity (*F*) between those cases who were homozygous versus those who were compound heterozygous among exome-positive cases, we performed logistic regressions in which the outcome was a dichotomous variable for homozygous versus compound heterozygous and the predictor was the *F* coefficient variable. The analysis was done with the overall sample population and was stratified by reported race/ethnicity.

Significance thresholds were adjusted using a Bonferroni correction: *P*-value < 0.008 for the analysis of ES diagnostic yield across six genetic ancestries (excluding Oceania due to insufficient cases) and *P*-value < 0.0071 for the analysis of consanguinity differences between homozygous and compound heterozygous exome-positive cases. The consanguinity analysis was conducted for the entire sample, excluding the few individuals with an *F* coefficient > 0.20, and by subgroup based on maternal-reported race/ethnicity (excluding Oceania, Middle Eastern, and Native American due to insufficient cases). These thresholds were calculated by dividing the original alpha level (0.05) by the number of tests performed—6 tests for diagnostic yield by genetic ancestry and 7 tests for the consanguinity analysis.

Note again that our variant annotation is different from that in other settings—for example, for rare diseases where the diagnosis is uncertain, and exome or genome sequencing is conducting across the entire exome/genome to identify potentially pathogenic variants explaining the patient's symptoms. Here, we only evaluate variants within a single gene or multiple genes associated with the presumed IEM diagnosis based on the MS/MS results and clinical follow-up. As mentioned above, this includes all protein-altering variants with a MAF < 0.5%. Many of the variants detected were novel^12^, and under other circumstances would likely be called VUS. Because loss of function is a mechanism of disease for these IEMs, the frameshift, stopgain, splice variants would plausibly be called P/LP, but probably far fewer of the missense variants (which were the majority) would be without clear functional data. But as described, this protocol was used to maximize sensitivity, at the possible expense of specificity. As previously reported, using more stringent criteria for variant annotation increased the specificity of 98.4% to 99.4% but drastically reduced the sensitivity to 55% from 88%^12^.

However, to specifically address the question of how much results would differ if we only included variants from the “curated arm” of our pipeline^12^ (i.e., were found in ClinVar and/or HGMD), we compared by *t*-tests the average ancestry of cases that were ES-diagnosed based only on curated variants (homozygous/hemizygous or compound heterozygous) versus those whose ES-diagnosis included non-curated variants.

## Supplementary information

Supplementary information

## Data Availability

The de-identified residual DBS from the California Biobank for this project (SIS request number 496) were obtained with a waiver of consent from the Committee for the Protection of Human Subjects of the State of California, under project no. 14-07-1650 and in compliance with CDPH Biospecimen/Data Use and Confidentiality Agreement. California blood specimens and any data derived from the newborn screening program are confidential and subject to strict administrative, physical, and technical protections. California law precludes any researcher from sharing blood specimens or uploading individual data derived from these blood specimens into any genomic data repository. Researchers desiring access to these data would need to make a separate application to the CPDH.
