# Supplementary material for: No association between genetic ancestry and exome sequencing-based diagnosis of inborn errors of metabolism
Source: NPJ Genom Med. 2026 Mar 27;11:27. doi: 10.1038/s41525-026-00562-3 (PMC13187471; doi:10.1038/s41525-026-00562-3)
Supplement: Supplementary file 1 — Supplementary information [file 41525_2026_562_MOESM1_ESM.pdf]

Figure S1: Visualization of Genetic Ancestry on PC1 and PC2 Axes

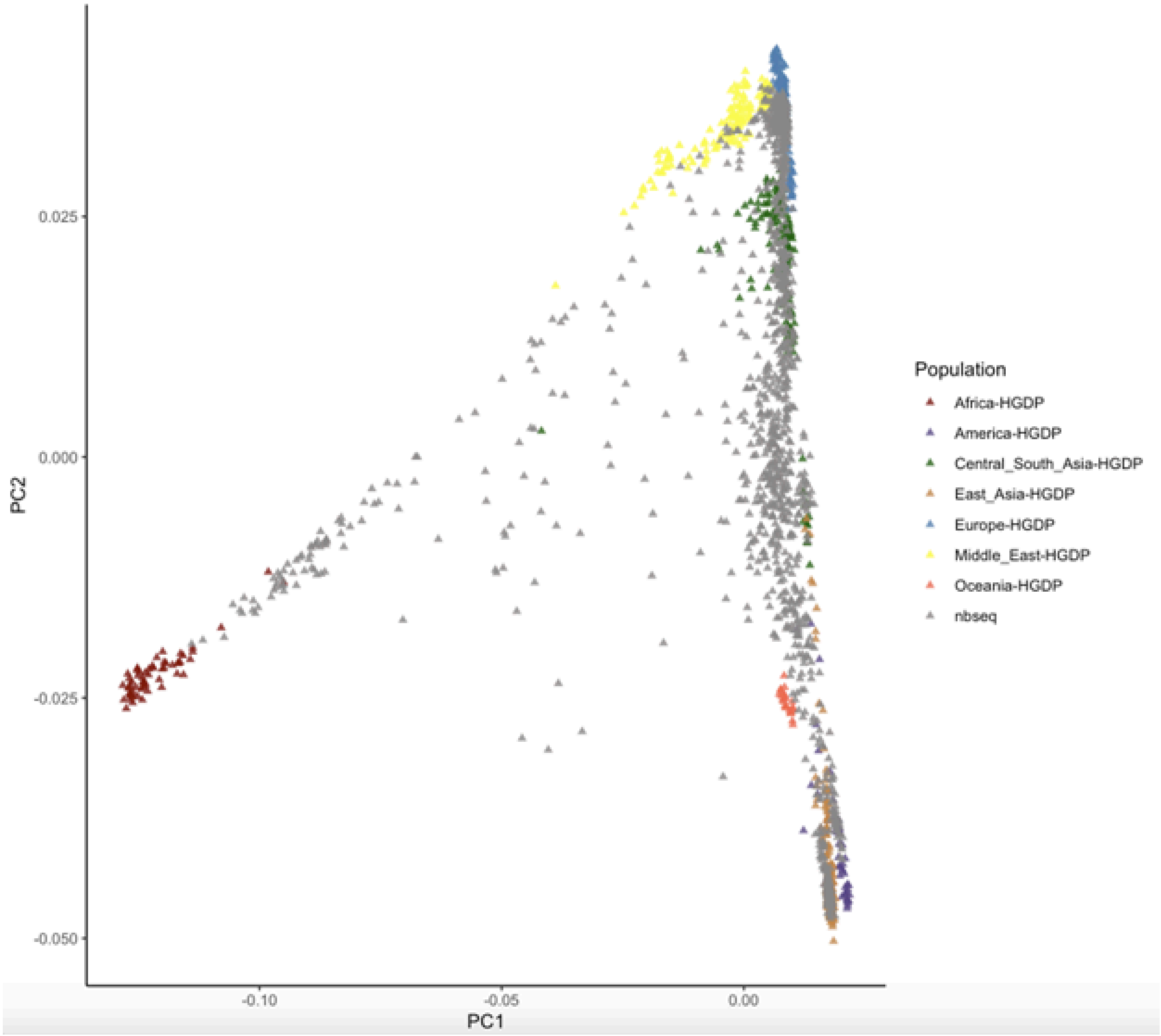

Visualization of genetic ancestry distributions along the first two principal components (PC1 and PC2), highlighting major axes of genetic variation across populations.

Figure S2: Visualization of Genetic Ancestry on PC3 and PC4 Axes

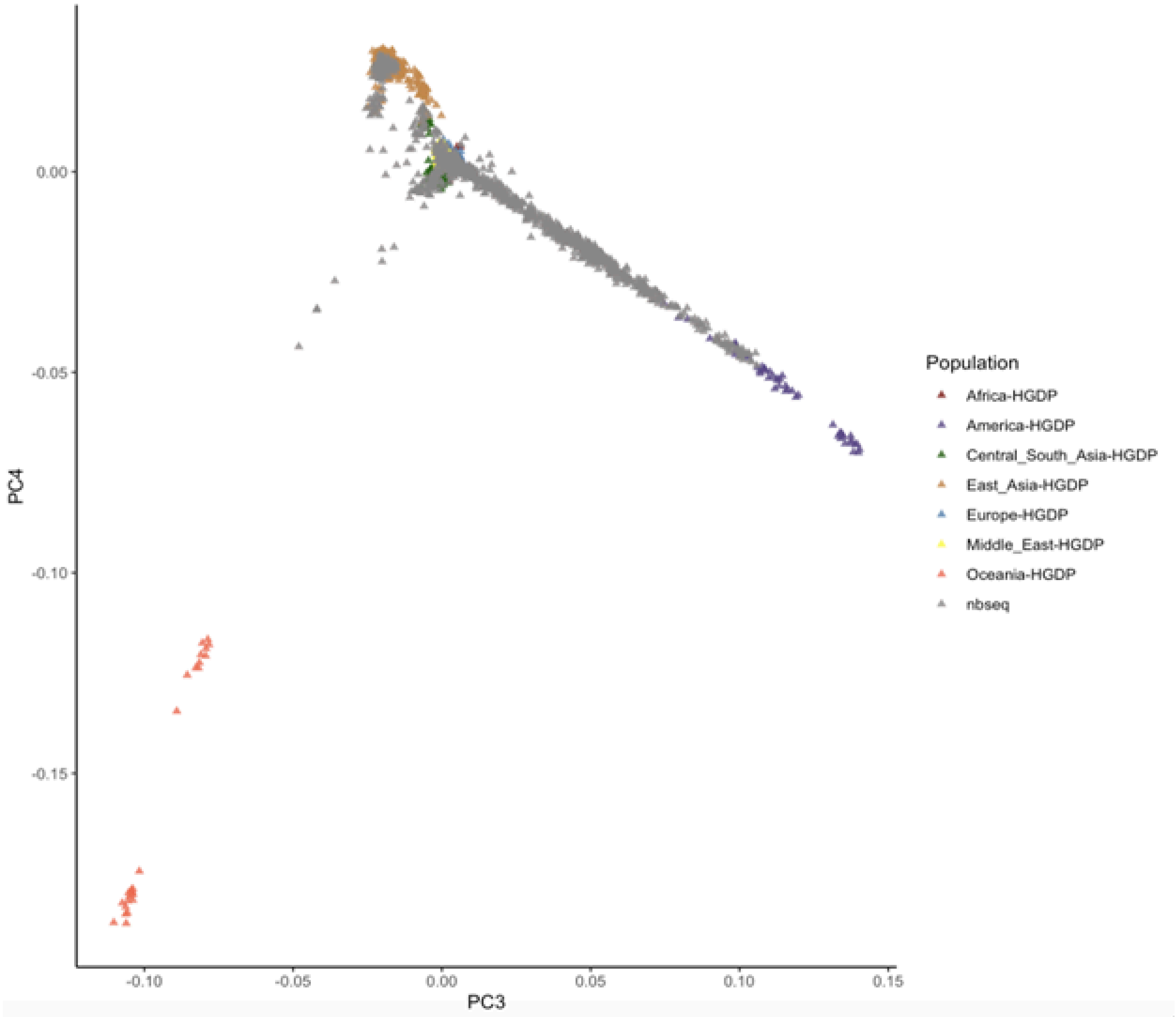

Visualization of genetic ancestry distributions along the third and fourth principal components (PC3 and PC4), distinguishing finer-scale population structure and genetic differentiation.

Figure S3. Distribution of Genetic Ancestries Across Cases

European Ancestry: Positive vs Negative Cases

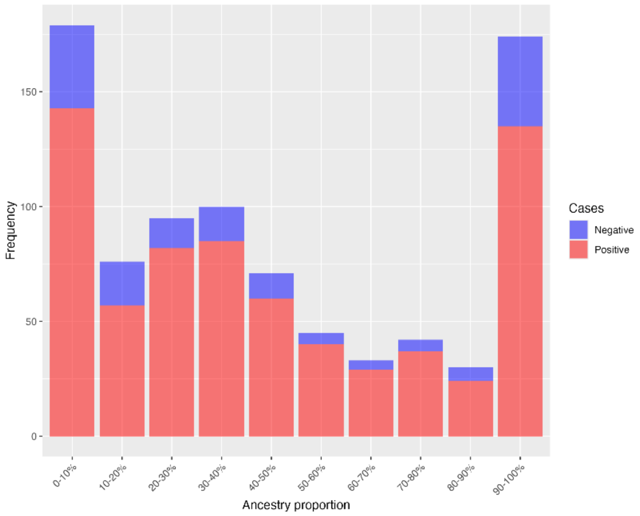

South Asian Ancestry: Positive vs Negative Cases

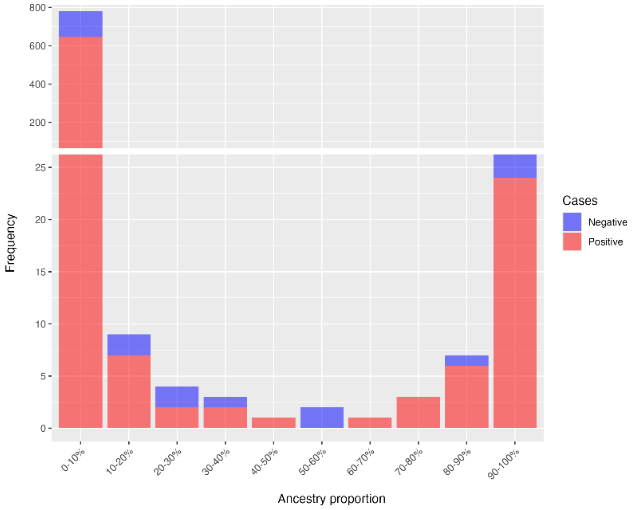

Middle East Ancestry: Positive vs Negative Cases

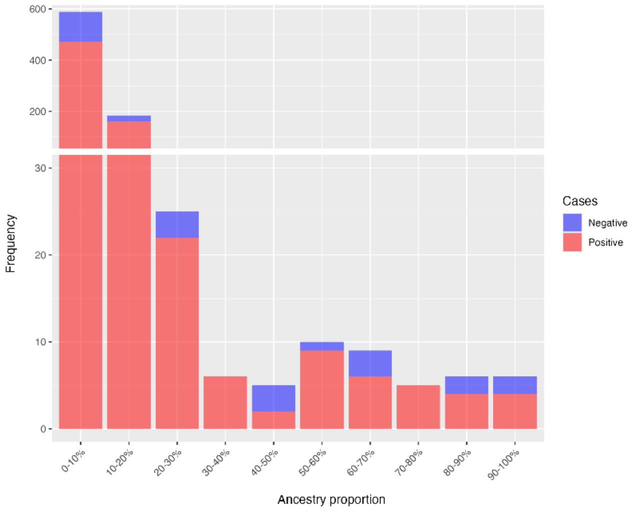

Native American Ancestry: Positive vs Negative Cases

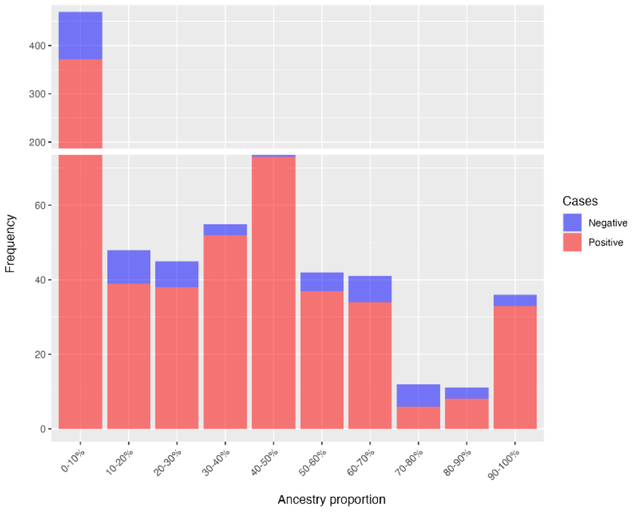

East Asian Ancestry: Positive vs Negative Cases

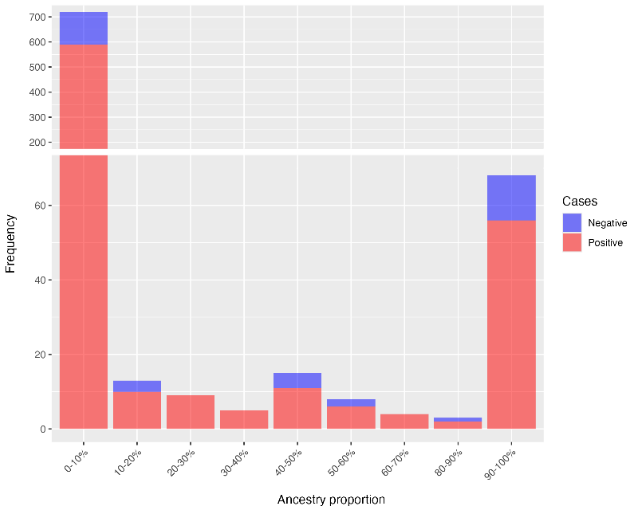

African Ancestry: Positive vs Negative Cases

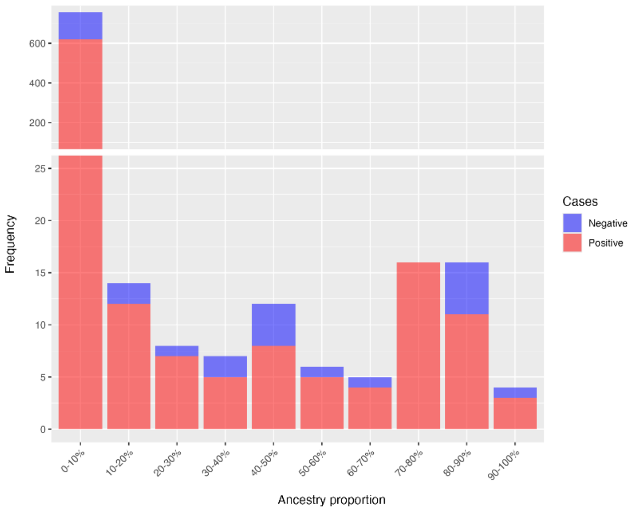

Figure S4. Distribution of F Coefficients Across Ancestries for Homozygosity and Compound Heterozygosity

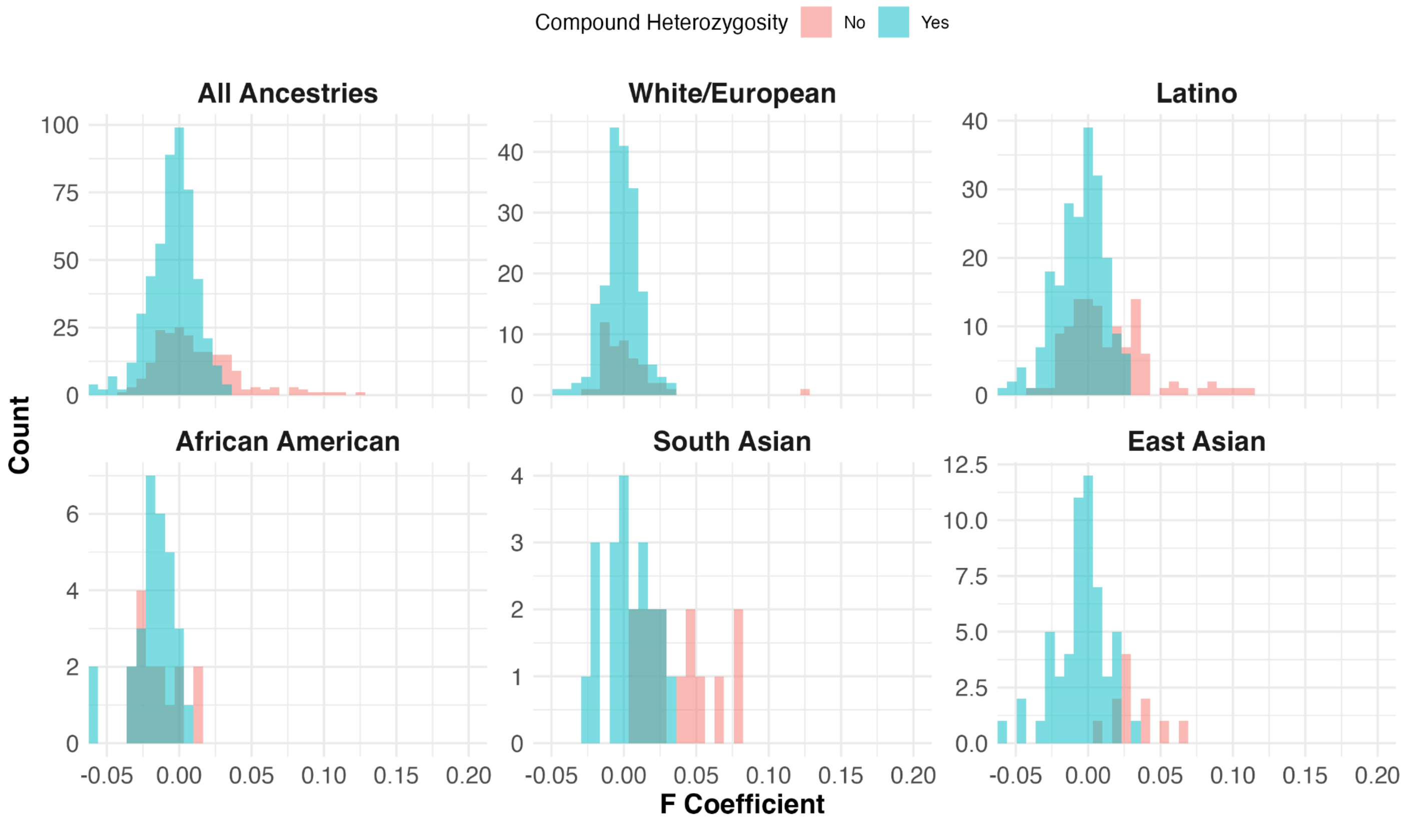

Table S1. Number of cases (%) in each bin and Cochran-Armitage trend test results

Ancestry	Case Type	0–12.5%	12.5–37.5 %	37.5–62.5 %	62.5–87.5 %	87.5–100 %	Z-statistic	P-value
**AFR**	Negative	137 (89.5%)	2 (1.3%)	7 (4.6%)	4 (2.6%)	3 (2.0%)	-0.62034	0.53504
**AFR**	Positive	626 (90.5%)	18 (2.6%)	15 (2.2%)	28 (4.0%)	5 (0.7%)	—	—

**AMR**	Negative	102 (66.7%)	14 (9.2%)	19 (12.4%)	14 (9.2%)	4 (2.6%)	—	—
**AMR**	Positive	380 (54.9%)	111 (16.0%)	125 (18.1%)	42 (6.1%)	34 (4.9%)	1.71974	0.08548
**EAS**	Negative	132 (86.3%)	2 (1.3%)	6 (3.9%)	0 (0.0%)	13 (8.5%)	—	—
**EAS**	Positive	592 (85.5%)	21 (3.0%)	18 (2.6%)	5 (0.7%)	56 (8.1%)	-0.03538	0.97178
**EUR**	Negative	39 (25.5%)	43 (28.1%)	43 (28.1%)	19 (12.4%)	9 (5.9%)	—	—
**EUR**	Positive	152 (22.0%)	190 (27.5%)	131 (18.9%)	75 (10.8%)	144 (20.8%)	-0.09854	0.92150
**MID**	Negative	123 (80.4%)	19 (12.4%)	5 (3.3%)	3 (2.0%)	3 (2.0%)	—	—
**MID**	Positive	524 (75.7%)	137 (19.8%)	15 (2.2%)	11 (1.6%)	5 (0.7%)	-0.14378	0.88568
**SAS**	Negative	136 (88.9%)	4 (2.6%)	2 (1.3%)	0 (0.0%)	11 (7.2%)	—	—
**SAS**	Positive	648 (93.6%)	9 (1.3%)	1 (0.1%)	8 (1.2%)	26 (3.8%)	-1.77976	0.07511

Table S2. F-score by Ancestry

Ancestry	Sample Size	Median F	IQR
EUR	386	-0.0026	-0.0120 – 0.0058

SAS	47	0.0157	0.0012 – 0.0379
MID	38	0.0022	-0.0093 – 0.0439
AMR	226	0.0190	-0.0107 – 0.0312
EAS	93	0.0000	-0.0103 – 0.0171
AFR	55	-0.0195	-0.0290 – -0.0091

Table S3. Kruskal-Wallis Test

**Test**	**Chi-Squared**	**df**	**p-value**
Kruskal-Wallis Rank Sum	84.995	5	<0.0001
